# On the Progress of the Variolæ Vaccinæ

**Published:** 1799-09

**Authors:** 


### Medical and Physical Journal.

VOL. II.] SEPTEMBER, 1799. [NO. VII.

### *On the Progrefs of the Variolæ Vaccinæ:* By *Dr.* PEARSON.

DR. PEARSON's communication of a paper, entitled "*A Statement of the Progrefs in the Practice of the* Vaccine Inoculation, *and* TRIALS *to determine fome important Facts relating to the Vaccine Difeafe,*" came much too late for infertion in the prefent Number.

We fhall now anticipate the accounts fo far as to acquaint our readers that the paper refpects—

1. The proportion of deaths by the vaccine difeafe to be one in about 2000 already inoculated, and in the fmall-pox the proportion is eftimated at ten times this number of deaths, or one out of 200.

2. That no eruptions like the fmall-pox have been feen by the author, or are ftated in the accounts fent to him during the laft four months.

3. That although in many cafes the cow-pox by inoculation is a more fevere difeafe, and attended with more alarming fymptoms than was originally reprefented, yet, in general, it is a far flighter difeafe than the inoculated fmall-pox.

4. That neither in his own practice, nor in that of his correfpondents, have any bad confequences enfued from the inflamed arms, although in fome cafes the eryfipelatous, or as Dr. Pearfon calls it, *erythematous* affections was very formidable. In particular he ftates, that the conftitution was very little difordered when almoft the whole arm was covered with erythema; and that this affection, without any applications, or merely fedative ones, fpeedily difappeared.

Dr. Pearfon has the fatisfaction of announcing, that the vaccine inoculation has occafioned a confiderable fenfation in Germany, and that fome of the principal phyficians at Vienna, viz. Dr. DE CARRO and Dr. FERRO, have already inoculated their own children with matter tranfmitted by himfelf:—that Dr. FRANK propofes to fubftitute the cow-pox for the fmall-pox in his inoculation-practice.

Dr. Pearſon expects ſoon to receive accounts from Portugal. In Scotland, nearly one hundred perſons have been inoculated by Dr. ANDERSON; and Dr. Pearſon finds that the practice is extending rapidly in that country.

But the moſt curious part of this paper, is that concerning his *trials* to obtain ſome uſeful determinations reſulting from experience.

1. It is known that the ſmall-pox renders the conſtitution unſuſceptible of this diſeaſe a ſecond time.

2. It ſeems now proved, that the cow-pox renders the conſtitution unſuſceptible of the ſmall-pox.

3. Dr. Pearſon ſhews by experiments, that perſons who have had the *ſmall-pox* cannot take the cow-pox, contrary to Drs. JENNER and WOODVILLE.

4. It might hence be concluded, that a perſon who has undergone the cow-pox is unſuſceptible of the cow-pox a ſecond time: but although the preceding propoſitions ſeem to demonſtrate this laſt fact, Dr. Pearſon does not truſt to reaſoning, but determines it by obſervations.

